# Isolation and characterization of marine microorganisms capable of degrading plastics

**DOI:** 10.1128/msystems.01388-25

**Published:** 2025-12-08

**Authors:** Qi Zeng, Liwen Chang, Yuqing Liu, Songbiao Shi, Jian Yang, Qinglian Li, Lijuan Long, Xinpeng Tian

**Affiliations:** 1 State Key Laboratory of Tropical Oceanography, Key Laboratory of Tropical Marine Bio-resources and Ecology, Guangdong Key Laboratory of Marine Materia Medica, RNAM Center for Marine Microbiology, South China Sea Institute of Oceanology, Chinese Academy of Sciences 74718 , Guangzhou, China; 2 University of Chinese Academy of Sciences, Beijing, China; CNRS Delegation Bretagne et Pays de Loire, Nantes, France

**Keywords:** plastic biodegradation, pure culture isolation, marine microbial resources, polyethylene terephthalate, polyurethane, polyethylene

## Abstract

The escalating crisis of global plastic pollution has prompted increasing efforts toward the identification of plastic-degrading microorganisms as environmentally sustainable solutions. Despite marine ecosystems being the most affected, the systematic exploration of culturable plastic-degrading microorganisms remains critically underexplored. Here, we established the largest marine microbial repository for plastic biodegradation, comprising 1,377 bacterial and 202 fungal strains isolated through optimized culture strategies targeting 13 plastic polymers. Methodological benchmarking revealed selective enrichment combined with carbon-supplemented dual-layer plating as the most effective isolation approach. Plastic substrate specificity drove phylogenetic divergence among degraders, with *Bacillus altitudinis* emerging as the dominant polyurethane (PU)-degrading lineage. Comprehensive functional screening identified five polyethylene terephthalate (PET)-degrading strains (*Cerrena* sp., *Hortaea werneckii*, *Streptomyces badius*, *Streptomyces cinereoruber*, *Cladosporium* sp.), five polyethylene (PE) degraders (*Qipengyuania citrea*, *Gordonia mangrovi*, *Penicillium oxalicum*, *Penicillium aethiopicum*, *Streptomyces pluricolorescens*), and 130 polyester-based PU-degrading strains spanning *Bacillus*, *Psychrobacter*, *Priestia*, and *Cladosporium* genera. This work provides both a foundational microbial resource for plastic biodegradation and establishes a methodological framework for the targeted isolation of plastic-degrading pure cultures.

**IMPORTANCE:**

Marine plastic pollution presents a significant global challenge, with millions of tons entering the oceans each year, threatening marine life and ecosystem integrity. Microbial degradation offers a promising, sustainable solution by leveraging natural biological processes to break down plastics. This study makes a substantial contribution to the field by systematically examining 13 plastic types and establishing the largest known marine microbial resource for plastic degradation. Over 1,500 bacterial and fungal strains were isolated from diverse marine environments through optimized culturing strategies. Key microorganisms capable of degrading commonly used plastics—such as polyethylene terephthalate (bottles), polyurethane (foams), and polyethylene (packaging)—were identified. These findings lay a critical foundation for the development of microbial-based technologies to mitigate plastic pollution, offering scalable and environmentally friendly solutions to protect marine ecosystems.

## INTRODUCTION

Plastics are synthetic polymers with exceptional durability, making them persistent pollutants in marine ecosystems (1). In marine environments, plastics are distributed across surface and deep waters, sediments (2), polar sea ice (3), and even the deepest ocean trenches (4). The massive accumulation of plastic waste not only threatens ecosystems and human health but also represents a significant loss of valuable resources (5). While plastic pollution has become a well-recognized global issue, the isolation of functional microorganisms capable of breaking down diverse polymers represents an important component in developing sustainable biodegradation strategies (6). Pure-culture isolation provides essential resources for mechanistic studies and forms the basis for advancing microbial biotechnology toward plastic recycling, as exemplified by the discovery of *Ideonella sakaiensis*, a model bacterium for polyethylene terephthalate (PET) degradation (7).

To date, most plastic-degrading strains have been isolated from terrestrial environments such as landfills, compost, and wastewater treatment plants, whereas marine ecosystems remain an underexploited reservoir. The unique conditions of marine habitats—low temperatures, high salinity, and elevated hydrostatic pressure—demand microbial resources specifically adapted to these environments (8). However, the isolation of such strains has been limited, contributing to a narrow representation of degraders, predominantly those targeting PET. Efficient degraders of other polymers, including polyethylene (PE), polypropylene (PP), and polyurethane (PU), remain scarce (9).

Conventional culture-based strategies for isolating plastic-degrading microorganisms remain underdeveloped and lack systematic evaluation. Most studies rely on a narrow set of media, such as minimal mineral salt medium (MSM) or Bushnell-Haas medium, with plastic polymers provided as the sole carbon source (10). The limited diversity of such isolation media may partly explain the low efficiency of current laboratory-based screening for plastic degraders. In addition to traditional solid-plate approaches, some studies have applied advanced techniques such as single-cell Raman spectroscopy, which enables *in situ* identification and cataloging of functional microorganisms without the need for pure cultures (11). Although powerful, this method requires costly instrumentation and complex procedures, limiting its accessibility for most laboratories. Therefore, there is a pressing need to optimize traditional plate-based isolation methods, retaining their advantages of simplicity and low cost while improving the efficiency of isolating and screening functional degraders.

**Fig 1:**
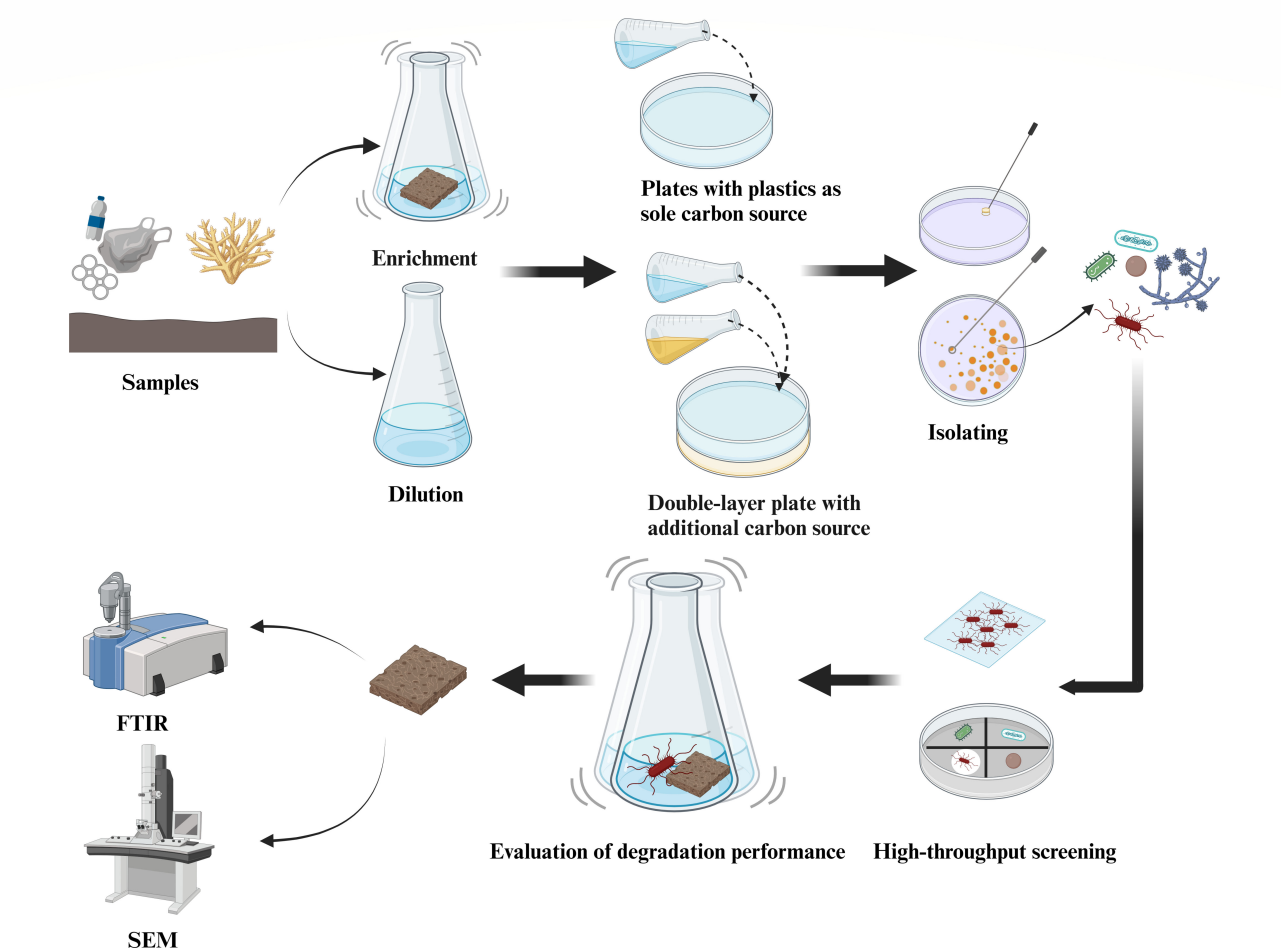
Workflow for the isolation and characterization of plastic-degrading microorganisms from marine environments. Environmental samples were subjected to enrichment or direct dilution, followed by isolation on plates with plastics as the sole carbon source or on carbon-supplemented double-layer plates. The obtained strains were purified and subjected to high-throughput screening for plastic-degrading activity. Degradation performance was further evaluated using physicochemical and morphological analyses, including Fourier transform infrared spectroscopy (FTIR) and scanning electron microscopy (SEM). This figure was created using Biorender.com.

To address these challenges, our study systematically evaluated different strategies for isolating and screening plastic-degrading microorganisms (Fig. 1). We compared enrichment versus direct dilution as initial sample treatments and tested the effects of using plastics as the sole carbon source versus carbon-supplemented media. Based on these comparisons, we established a repository of marine plastic-degrading bacteria and fungi across multiple polymer types. This work provides both a methodological framework for the targeted isolation of plastic-degrading microorganisms and a valuable microbial resource for advancing future research in plastic biodegradation.

## MATERIALS AND METHODS

### Sample collection and pretreatment

Plastic waste and sediment samples were collected from the mangroves and sandy beaches of Qi’ao Island, Zhuhai City, China, in January 2023. Coral samples were obtained from islands and reefs in the South China Sea in January 2021, and surface sediment samples were taken from the Pearl River estuary in March 2021. Additionally, deep-sea sediment samples were collected from the South China Sea in May 2022 (Table S1). All samples were placed in a sterile bottle and stored at −20°C until further processing.

For sample pretreatment, both direct-dilution and enrichment methods were used. In the direct-dilution method, samples were diluted in sterile 1× PBS buffer to concentration ratios of 10:1 vol/vol and 100:1 vol/vol by successively diluting 1 g of each sample. For the enrichment method, 1 g of each sample was added to enrichment media containing plastic as the primary carbon source (2 × 2 cm plastic film, 0.1% rhamnolipid, minimal mineral salt medium [MSM]). The samples were incubated at 150 rpm and 28°C for 30 days, with 5 mL of fresh enrichment media added every 15 days.

### Preparation of selective isolation media

Two types of specialized selective isolation media, designated as type A and type B, were developed. Type A medium consisted of a basal MSM with 1.5% agar, a mixture of 50% natural seawater and 50% double-distilled water, and 1.0% (wt/vol) plastic as the carbon source. Type B medium was structured as a two-layer plate: the lower layer contained 10% 2216E marine broth, while the upper layer consisted of type A medium.

Thirteen different polymers were selected as carbon sources, including 12 solid microplastics and 1 aqueous polyurethane. The 12 solid microplastics were high-density polyethylene (HDPE), low-density polyethylene (LDPE), polyamide 6 (PA6), polyamide 66 (PA66), polybutylene adipate terephthalate (PBAT), polycaprolactone (PCL), PET, polylactic acid (PLA), PP, polystyrene (PS), polyvinyl alcohol (PVA), and polyvinyl chloride (PVC), all obtained from DuPont Co., Ltd. The aqueous PU (Impranil DLN) was acquired from Covestro Co., Ltd. All solid polymer samples were sterilized with 70% ethanol for 24 hours and then dried at 30°C. The aqueous polyurethane was sterilized using 0.22 µm syringe filters from Pall Co., Ltd.

### Isolation, maintenance, and identification of strains

A 200 µL suspension from the different sample pretreatment methods was spread onto the selective isolation media and incubated at 28°C for 21 days. Distinct colonies were then isolated and cultivated on either 2216E or Potato Dextrose Agar media at 28°C. For long-term preservation, the pure cultures were stored in 30% (vol/vol) glycerol suspensions at −80°C.

For genomic analysis, bacterial and fungal DNA were extracted using the Chelex-100 and cetyltrimethylammonium bromide methods, respectively. The 16S rRNA genes or the ITS regions were amplified according to the protocols described by Ramdass et al*.* (12). Strain identification and pairwise gene sequence similarity calculations were performed using the EzBioCloud database (https://www.ezbiocloud.net/) for the 16S rRNA gene sequences and the NCBI BLAST tool (13) for ITS sequences.

### Phylogenetic analysis and tree visualization

Phylogenetic relationships of representative plastic-degrading isolates were inferred using 16S rRNA gene sequences (for bacteria) and ITS sequences (for fungi). For taxa with multiple strains, one representative sequence per species was selected to avoid redundancy. Sequences were aligned using MUSCLE (14) implemented in MEGA X (15), and a neighbor-joining tree was constructed with 1,000 bootstrap replicates to assess branch support. The resulting tree was visualized and annotated in the Interactive Tree of Life platform (https://itol.embl.de/) (14, 16), where outer rings were used to indicate phylum-level affiliations, plastic types of origin, and substrate-specific enrichment patterns.

### Screening of potential plastic-degrading microorganisms

Preliminary screening methods included microbial adhesion growth observation and extracellular enzyme activity assays. For microbial adhesion growth observation, isolated strains were incubated in MSM containing plastic films at 28°C, 150 rpm for 40 days. Strains displaying notable adhesion and proliferation on the plastic films were identified as potential candidates for plastic degradation.

The enzyme activity assays focused on extracellular enzymes such as laccase, ligninase, lipase, polyurethanase, and cellulase. Laccase, lipase, and polyurethanase are well-known plastic-degrading enzymes. Given the structural similarities between lignin, cellulose, and plastics, screening for lignin peroxidase and cellulase activities may also help identify potential plastic-degrading microorganisms. Specific substrates such as guaiacol, lignin, tributyrin, Impranil DLN, and cellulose were used to evaluate the activities of laccase, lignin peroxidase, lipase, polyurethanase, and cellulase, respectively (17). Detailed descriptions of the media used for these tests are provided in Table S2 of the appendix.

### Evaluation of degradation characteristics

The activity of *Bacillus altitudinis* strains toward PU was quantitatively assessed using the halo index (HI). For HI determination, isolates were cultivated on modified solid MSM plates supplemented with 3% PU and 0.5% peptone. After incubation, images of the colonies and their surrounding transparent zones were captured and analyzed with Fiji (18). The areas of the colonies and halos were measured using the freehand selection tool, and the HI was calculated as the ratio of halo area to colony area (HI = halo area / colony area). Each strain was analyzed in triplicate, and mean values with standard deviations were obtained.

Strains identified as potential plastic degraders were cultivated in MSM containing 2 × 2 cm plastic films and incubated at 28°C, 150 rpm for 30 days. After incubation, the biodegradation of the plastic films was characterized using scanning electron microscopy (SEM) and Fourier transform infrared spectroscopy (FTIR). SEM was used to observe microbial colonization and plastic’s morphological changes. Plastic films with biofilms were initially fixed with 5% glutaraldehyde, dehydrated in a graded ethanol series (30%–100%) for 20 minutes each, and then subjected to critical-point drying with CO_2_. The biofilms were removed from the plastic surfaces using an ultrasonic cleaner with 1% SDS, distilled water, and 75% ethanol. The cleaned films were dried at 35°C and sputter-coated with a thin layer of gold and platinum (10 nm) using a Hitachi MC1000 Ion Sputter. Observations were made with a field emission scanning electron microscope (Hitachi S-3400 N, Japan) at an accelerating voltage of 10 kV. FTIR analysis was conducted to detect changes in the functional groups of the plastics. Plastic films without biofilms were analyzed in the wavelength range of 400 cm^−1^–4,000 cm^−1^ at a resolution of 1 cm^−1^ using a Nicolet-360 FTIR spectrometer (Waltham, USA). Each spectrum was obtained by averaging 35 scans.

## RESULTS AND DISCUSSION

### Diversity of potential plastic-degrading microorganisms

**Fig 2:**
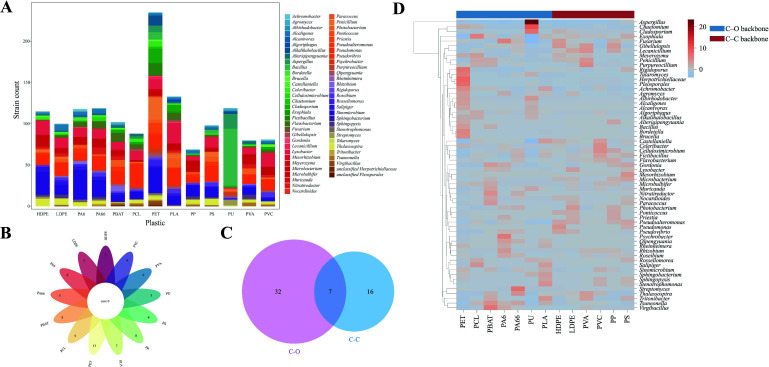
Microbial genera isolated from various plastic surfaces and their enrichment across plastic types. (**A**) Stacked bar chart representing the genera of microorganisms isolated from different plastic types, excluding taxa with abundances lower than 0.15%. (**B**) Petal diagram illustrating the unique microbial genera significantly enriched on 13 distinct plastics, highlighting the variation in genera across plastic types. (**C**) Venn diagram displaying the overlap of microbial genera significantly enriched on plastics with C-O and C-C backbones, indicating both shared and distinct genera between the two categories. (**D**) Heatmap of standardized residuals showing the preferences of various microbial genera for specific plastic types, excluding taxa with abundances lower than 0.1%.

Using 13 types of plastics as carbon sources, we isolated a total of 1,579 strains, comprising 1,377 bacterial strains and 202 fungal strains. The highest number of isolates (305 strains) was obtained from PET plates (Fig. 2A). Plastics were categorized into two groups: (i) polymers with carbon-oxygen (C-O) linkages in the main chain (e.g., PET, PCL, PBAT, PA6, PA66, PU, PLA), which yielded 1,022 strains; and (ii) polymers with pure carbon-carbon (C-C) backbones lacking heteroatoms (e.g., HDPE, LDPE, PVA, PVC, PP, PS), which yielded 557 strains (Fig. S1). The higher count of isolates from C-O backbone plastics likely reflects the susceptibility of hydrolysable ester bonds to microbial attack, whereas the more stable C-C backbones resist biodegradation and degrade mainly through oxidative processes.

At the genus level, *Flavobacterium*, *Pseudomonas*, *Sinomicrobium*, *Microbacterium*, and *Lysobacter* were isolated from all 12 solid microplastics. Among these, *Sinomicrobium* (192 isolates, 12.2%), *Pseudomonas* (180 isolates, 11.4%), and *Lysobacter* (151 isolates, 9.6%) were the most abundant. Many *Pseudomonas* species are metabolically versatile and have been reported to degrade multiple plastics, including PE, PP, PVC, PS, PU, PET, polyethylene succinate, polyethylene glycol, and PVA at different levels of efficiency (19). At the species level, *Sinomicrobium oceani* (192 isolates, 12.2%) was the most frequently isolated, known for its ability to degrade alginate (20). *Lysobacter maris* (126 isolates, 8.0%) was also frequently isolated, known for its rich content of chitin- and cellulose-degrading enzymes (21, 22). *Pseudomonas khazarica* (124 isolates, 7.9%) was recognized for its ability to degrade PAHs (23).

To further assess relationships between plastic types and microbial diversity, a χ^2^ test of independence was performed. The contingency analysis (Table S3) confirmed that plastic type significantly influenced microbial distribution (χ² = 2,293.27, *P* < 0.001). Standardized residuals were then calculated to evaluate microbial preferences for different plastics, where a residual >2 indicates a significant enrichment of certain microbes on specific substrates (Table S4). The analysis revealed that none of the 13 plastics shared a common significantly enriched genus (Fig. 2B; Table S5). When plastics were grouped by backbone types, these genera, including *Pseudoalteromonas*, *Chaetomium*, *Photobacterium*, *Sinomicrobium*, *Nitratireductor*, *Alteriqipengyuania*, and *Lysobacter,* were significantly enriched on both C-O and C-C groups (Fig. 2C), suggesting that these taxa possess versatile hydrolytic and oxidative enzyme systems enabling broad substrate degradation.

For C-O type plastics, *Cladosporium* showed a strong preference for PET and PLA (Fig. 2D). Various *Cladosporium* strains have been reported to degrade PU and PET, such as *Cladosporium* sp. L-5 (24), *C. halotolerans* 6UPA1 (25), *C. cladosporioides* 156.01 (26), and *C. cladosporioides* CBMAI 2075 (27). These underscore the *Cladosporium* genus members’ potential to effectively break down plastics that contain C-O bonds. In addition, *B. altitudinis* demonstrated pronounced substrate specificity toward PU, accounting for 40.8% (Fig. S2) of isolates forming halos on PU plates.

For C-C type plastics, *Microbacterium* was significantly enriched on PVC and PP plates (Fig. 2D). *Microbacterium esteraromaticum* has been reported to degrade PS and PE through manganese peroxidase and lipase activity (28), supporting its potential role in C-C plastic degradation. *Microbulbifer* was enriched on PVA, PP, and HDPE plates, consistent with previous reports of *M. hydrolyticus* degrading LDPE (29). Similarly, *Thalassospira* was significantly enriched on PS and HDPE, and its strains have been reported to degrade PVA, phthalates, and PS (30–32), demonstrating broad degradation spectrums.

### Phylogenetic distribution and functional clustering

**Fig 3:**
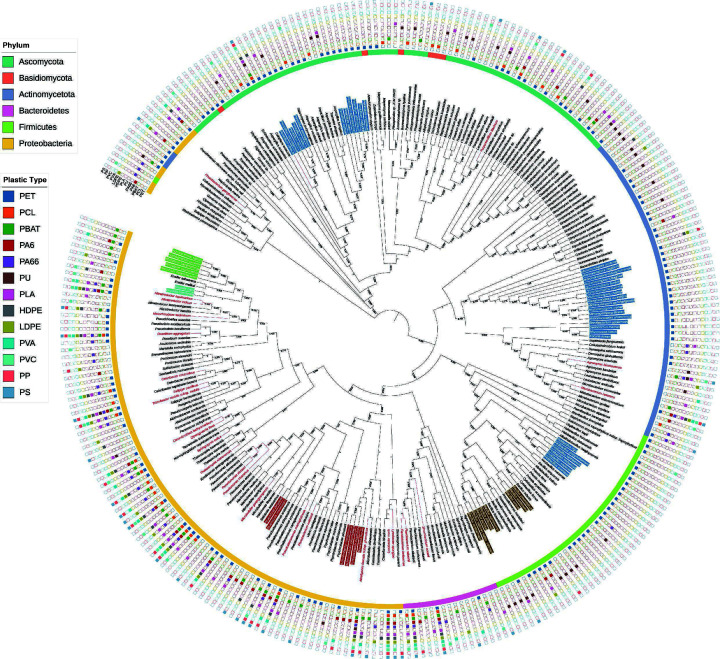
Phylogenetic distribution of plastic-degrading microorganisms isolated from 13 types of plastics. The neighbor-joining phylogenetic tree was constructed using 16S rRNA gene sequences (for bacteria) and ITS sequences (for fungi) from representative isolates, with one sequence selected per species when multiple strains were obtained. Bootstrap values were calculated from 1,000 replicates and are indicated at branch nodes. Outer colored bars denote phylum-level affiliations. Squares surrounding the tips indicate the plastic types from which each isolate was obtained. Labels highlighted in red represent taxa isolated from more than six different plastics, spanning both C-O and C-C backbones. Label backgrounds filled with colors corresponding to plastic types indicate taxa showing enrichment or preference for specific plastics.

To investigate the evolutionary relationships among plastic-degrading microorganisms, we constructed a neighbor-joining phylogenetic tree of all 1,579 isolates, with each strain annotated by its corresponding plastic source (Fig. 3). These isolates were distributed in six major phyla: *Proteobacteria*, *Firmicutes*, *Bacteroidetes*, *Actinomycetota*, *Ascomycota*, and *Basidiomycota*, with *Proteobacteria* and *Actinomycetota* being the most abundant.

At the genus and species levels, certain genera such as *Sinomicrobium*, *Flavobacterium*, *Pseudomonas*, and *Nitratireductor* were consistently recovered from more than six different types of plastics, encompassing both C-O and C-C backbones. In contrast, several clades exhibited strong substrate-specific preferences. For example, *Penicillium*, *Fictibacillus*, and *Micromonospora* were predominantly isolated from PET, consistent with their reported ability to hydrolyze ester linkages. *Bacillus* species formed a distinct PU-associated lineage, while *Achromobacter* and *Alcanivorax* were preferentially recovered from PA6. Similarly, *Brucella* and *Agrobacterium* showed enrichment on PVC and PBAT, respectively, reflecting narrow specialization toward these substrates. In addition, *Streptomyces* and *Cladosporium* were primarily associated with C-O backbone plastics (e.g., PET and PLA), suggesting evolutionary adaptation toward hydrolysable ester bonds.

Taken together, the phylogenetic framework revealed two major ecological strategies: (i) broad-spectrum generalists, such as *Sinomicrobium*, *Flavobacterium*, and *Pseudomonas*, which occur across diverse plastic types, and (ii) substrate specialists, which cluster according to specific plastic polymers or backbone chemistries, such as the preferences of *Micromonospora* for PET and *Bacillus* for PU.

### Effects of different isolation techniques

#### Impact of sample pretreatment

Both direct-dilution and enrichment methods were utilized for sample treatment. Enrichment led to significant microbial adhesion to plastic films (Fig. S3). After 30 days of incubation at 28°C, distinct plastic-associated taxa were recovered. On PET film, a brown colony of *Cladosporium* sp. SCSIO 81130 was isolated, a genus well known for PU degradation (33). In the H2 sample enriched with PVC film, a black fungal colony was isolated and identified as *Exophiala aquamarin*a SCSIO 81122. Related species such as *Exophiala oligosperma*, found in biofouled plastic textiles, carry genes associated with the degradation of hydrocarbons and plasticizers (34). On PP film, enrichment yielded gray colonies of *Algoriphagus zhangzhouensis* SCSIO 82448 and *Sphingopyxis indica* SCSIO 82452, both genera previously associated with hydrocarbon metabolism. Moreover, enrichment on PET film from the H4 sample produced a consortium including *Pseudoalteromonas arabiensis* SCSIO 82442, *Brevundimonas albigilva* SCSIO 82443, *Mycolicibacterium fortuitum* subsp. acetamidolyticum SCSIO 82444, and *Pseudomonas gallaeciensis* SCSIO 82447 (Table S6). Notably, *M. fortuitum* is adept at lignocellulose decomposition (35), while *P. gallaeciensis* has been isolated from oil-contaminated environments (36), both highlighting their potential for plastic degradation. It is widely recognized that adhesion is the first step in microbial plastic degradation, as it reduces the plastic’s surface hydrophobicity and promotes biofilm formation (37). Consistent with this, strains enriched from biofilm-attached communities in our study are likely candidates for plastic degradation. In addition, extended enrichment using plastics as the sole carbon source has also been shown to be essential for obtaining culturable plastic-degrading strains, as demonstrated in LDPE-degrading consortia that emerged only after 105 days of sequential enrichment (38).

Comparison of pretreatment strategies revealed significant differences in the isolated microbial communities. Enrichment yielded taxa frequently reported as plastic degraders, whereas these genera were not obtained from the direct-dilution isolation method. For example, enrichment of sample H1 favored *Pseudomonas* and *Thalassospira* (Table S7), both known for plastic degradation (30–32). Other enrichment-dominant genera, including *Celeribacter*, *Pseudoalteromonas*, and *Tsuneonella*, were also dominant in the enriched method, yet were rarely isolated using the direct-dilution isolation method.

#### Influence of isolation media

Type A medium consists of MSM supplemented with plastic as the sole carbon source, while type B medium includes an additional bottom layer of 2216E to provide supplementary nutrients. Type B showed better diversity at the genus level (57 vs 53 genera; Fig. S4) and yielded more isolates overall (Table S8) than type A. For example, sample H1 produced 196 colonies on type B plates compared to 141 colonies on type A, and ST1 yielded 60 colonies versus 36 colonies, respectively. These results indicate that the double-layer design of type B facilitates the recovery of a broader range of microbial communities, including potential degraders that may function synergistically in plastic biodegradation. While oligotrophic media such as MSM, Bushnell-Haas, and carbon-free basal medium are traditionally employed for isolating degraders, our findings suggest that the inclusion of minimal additional nutrients can enhance cultivability and improve the likelihood of recovering plastic-degrading strains. Future studies should evaluate a broader range of media formulations and nutrient supplements to optimize isolation strategies for plastic-degrading strains.

### Screening of potential plastic-degrading microorganisms

In the initial screening, various microorganisms were inoculated into liquid media with plastic films. Strains exhibiting adherence and growth on film surfaces were identified as potential plastic-degrading microorganisms. Fungi and actinomycetes—particularly *Cladosporium*, *Penicillium*, and *Streptomyces*—showed strong colonization ability (Table S9). Extracellular enzymatic activities were further evaluated using plate assays for laccase, ligninase, lipase, polyurethanase, and cellulase. Strains that exhibited a range of extracellular enzyme activities were considered promising candidates for plastic biodegradation (Table S10). Bacterial genera showing strong enzyme activities included *Bacillus*, *Flavobacterium*, *Lysobacter*, *Microbacterium*, *Microbulbifer*, *Photobacterium*, *Pseudomonas*, *Salipiger*, *Sinomicrobium*, and *Streptomyces*, while fungi primarily belonged to *Aspergillus*, *Chaetomium*, *Cladosporium*, and *Penicillium*. Notably, many of these genera have been previously reported to degrade compounds structurally analogous to plastics—such as cellulose, lignin, petroleum hydrocarbons, and plastic additives—supporting the reliability of this enzyme-based prescreening approach.

Most high-crystallinity plastics, including inherently biodegradable polymers such as PLA, rarely produce visible clearance zones on assay plates. Therefore, developing reliable methods to visualize biodegradation is crucial. In this study, we used substrates capable of producing either color changes or clearance zones, including guaiacol plates for laccase, Remazol Brilliant Blue R plates for ligninase, tributyrin emulsions for lipase, Impranil DLN plates for polyurethanase, and cellulose–Congo red plates for cellulase. In addition, previous studies have demonstrated that substrates such as bis(2-hydroxyethyl) terephthalate and ferulate can also generate visible halos, thereby enabling the identification of PET-degrading microorganisms (39). Collectively, the use of such indicator substrates improves screening efficiency and provides a practical strategy for discovering novel plastic-degrading microbes.

### Degradation performance of plastic-degrading microorganisms

#### Degradation of polymers with C-O backbones

**Fig 4:**
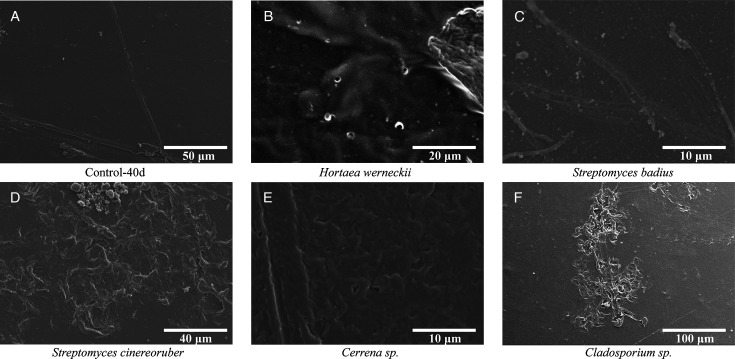
SEM images of PET films after 40 days of incubation under different microbial treatments. (**A**) Control film incubated in MSM without microbial inoculation (Control-40d). (**B**) *Hortea werneckii* SCSIO 81081. (**C**) *Streptomyces badius* SCSIO 80773. (**D**) *Streptomyces cinereoruber* SCSIO 80706. (**E**) *Cerrena* sp. SCSIO 81031. (**F**) *Cladosporium* sp. SCSIO 81078.

Among C-O backbone plastics, five strains exhibited pronounced degradation of PET. SEM observations showed that *Hortaea werneckii* SCSIO 81081, *Streptomyces badius* SCSIO 80773, *Streptomyces cinereoruber* SCSIO 80706, *Cerrena* sp. SCSIO 81031, and *Cladosporium* sp. SCSIO 81078 caused pronounced deterioration of PET films, with surface morphology markedly different from the linear scratches observed in controls (Fig. 4). These results suggest that these strains are capable of directly attacking PET surfaces. Supporting this, previous reports have shown that *Cerrena* is known to produce laccase that degrade plant polymers such as lignin (40, 41). *H. werneckii* has been reported to secrete hydrolytic enzymes including lipase, cellulase, and amylase (42, 43). *S. badius* can also produce cutinases, cellulase, and amylases (44), which enable the degradation of plastic analogs such as cellulose, starch, and lignin (45). Although reported in other contexts, these enzymatic capabilities support the potential involvement of these strains in ester bond cleavage and PET breakdown observed in this study.

**Fig 5:**
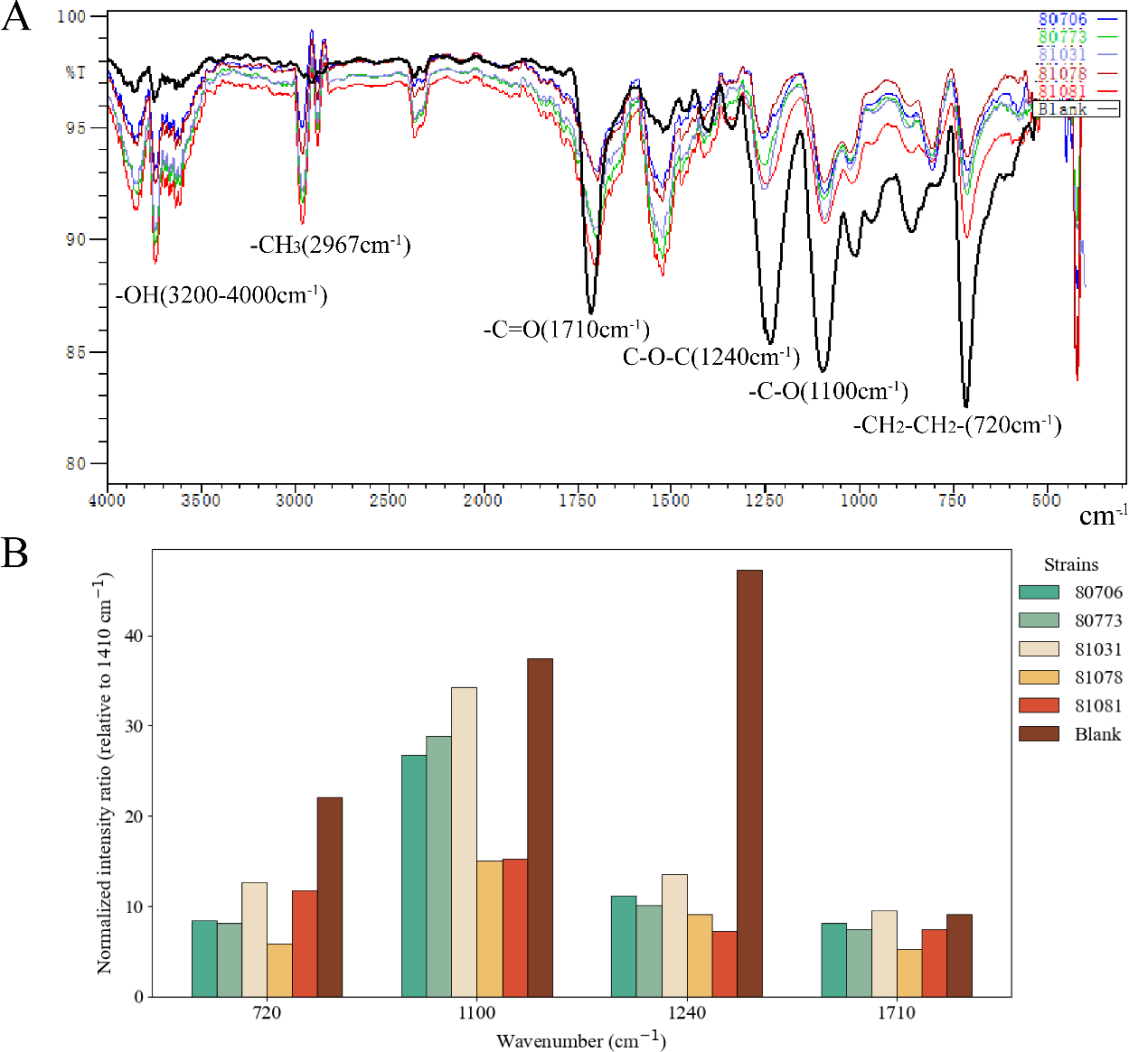
FTIR analysis of PET films after 40 days of incubation. The control film was incubated in MSM without microbial inoculation (Control-40d). Experimental groups included films incubated with *H. werneckii* SCSIO 81081, *S. badius* SCSIO 80773, *S. cinereoruber* SCSIO 80706, *Cerrena* sp. SCSIO 81031, and *Cladosporium* sp. SCSIO 81078. (**A**) Representative FTIR spectra of PET films incubated with different microbial strains compared to the blank control. Characteristic peaks associated with –OH (3,200 cm⁻¹–4,000 cm⁻¹), –CH_3_ (2,967 cm⁻¹), –C = O (1,710 cm⁻¹), C-O-C (1,240 cm⁻¹), C-O stretching (1,100 cm⁻¹), and –CH_2_–CH_2_- rocking (720 cm⁻¹) vibrations are indicated. (**B**) Quantitative comparison of normalized intensity ratios of characteristic absorption bands relative to the 1,410 cm⁻¹ reference band. Significant reductions were observed in the carbonyl (1,710 cm⁻¹), ester (1,240 and 1,100 cm⁻¹), and aromatic (720 cm⁻¹) regions across microbial treatments.

To further assess the extent of degradation, FTIR analyses were performed, providing detailed insights into the chemical changes occurring in the PET structure due to microbial degradation. In addition to qualitative spectral comparisons, quantitative analysis of peak areas was conducted after baseline correction (Fig. 5). The results showed that the characteristic absorption peaks at 720 cm⁻¹, 1,100 cm⁻¹, and 1,240 cm⁻¹, corresponding to the out-of-plane bending vibration of aromatic C-H and ester linkages in PET, exhibited pronounced reductions in the biodegraded samples compared to the blank. Specifically, the relative peak area at 1,100 cm⁻¹ decreased by 28.6%–85.5% across the experimental groups, while the 1,240 cm⁻¹ peak was reduced by 71.3%–84.7%, indicating substantial cleavage of ester bonds within the PET backbone. Similarly, the 720 cm⁻¹ peak, associated with aromatic C-H bending, decreased by 42.8%–73.8% relative to the control, further supporting degradation of the aromatic moieties. Moreover, the single absorption band at 1,710 cm⁻¹ in the control samples split into multiple smaller peaks in the degraded PET films, confirming structural destabilization of ester and aromatic groups. In addition, new absorption bands at 2,880 cm⁻¹ and 2,967 cm⁻¹ were detected in the experimental groups, corresponding to aliphatic –CH₂ and –CH₃ stretching, which indicates depolymerization of PET into simpler fragments. Furthermore, an increased absorbance in the 3,500–3,950 cm⁻¹ region was observed, corresponding to O-H stretching vibrations, and consistent with hydrolytic cleavage of ester linkages. Taken together, both the quantitative reduction in characteristic peak areas and the emergence of new spectral features provide strong evidence of PET polymer degradation induced by microbial activity. 

In addition to PET, a total of 130 strains were identified as capable of degrading polyester-based PU (Impranil DLN), most of them derived from the genera *Bacillus*, *Psychrobacter*, *Priestia*, *Penicillium*, and *Cladosporium* (Table S11). These strains produced clear zones on plates containing Impranil DLN as the sole carbon source (Fig. S5). Notably, many of these genera were also enriched on PET plates, suggesting strong esterase activity capable of cleaving ester bonds in both polyester-based PU and PET, thereby facilitating the degradation of C-O backbone plastics. Among the PU-degrading strains, *B. altitudinis* accounted for 41%. To further evaluate the PU-degrading performance of these isolates, their activity was quantified using the HI—the ratio of the clear zone area to the colony area—formed on PU plates (Fig. S6). Among the 53 isolates, considerable variation in PU degradation efficiency was observed, with strains SCSIO 82409 and SCSIO 82407 showing the strongest activity, exhibiting HI values up to 9.7. These highly active strains provide valuable biological resources for future studies on PU biodegradation.

#### Degradation of plastics with C-C backbones

**Fig 6:**
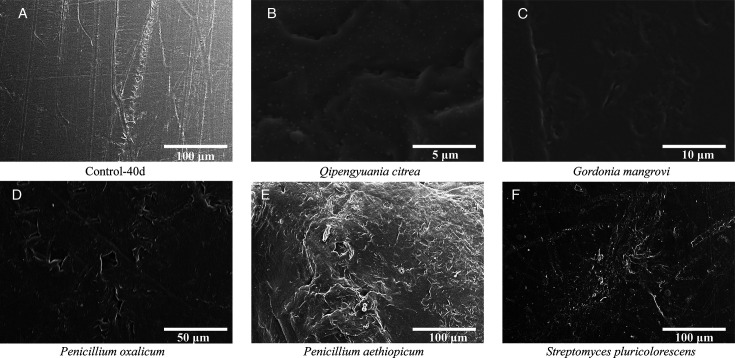
SEM images of PE films after 40 days of incubation under different microbial treatments. (**A**) Control film incubated in MSM without microbial inoculation (Control-40d). (**B**) *Qipengyuania citrea* SCSIO 80490. (**C**) *Gordonia mangrovi* SCSIO 80507. (**D**) *Penicillium oxalicum* SCSIO 81005. (**E**) *Penicillium aethiopicum* SCSIO 81068. (**F**) *Streptomyces pluricolorescens* SCSIO 80745.

In the investigation of degraders for plastics with carbon-carbon (C-C) backbones, five strains were identified as capable of degrading PE. SEM observations showed that *Qipengyuania citrea* SCSIO 80490, *Gordonia mangrovi* SCSIO 80507, *Penicillium oxalicum* SCSIO 81005, *Penicillium aethiopicum* SCSIO 81068, and *Streptomyces pluricolorescens* SCSIO 80745 induced marked deterioration of PE films, compared with the control group incubated without microbial inoculation (Fig. 6). Control films displayed regular, elongated scratches attributed to abiotic physical stress, whereas PE films exposed to the five strains exhibited irregular pits and cavities, suggesting direct microbial attack on the polymer surface.

**Fig 7:**
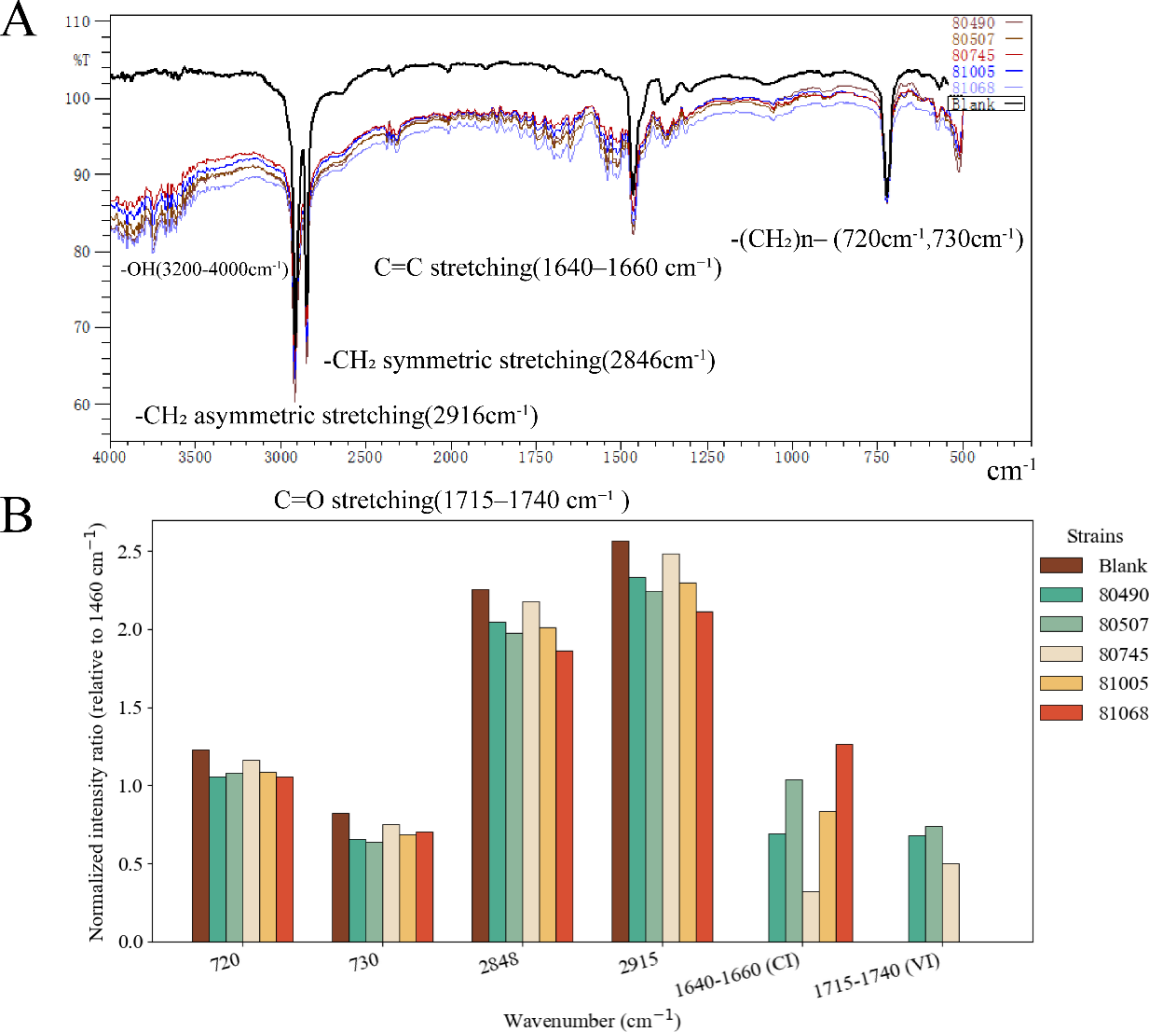
FTIR analysis of PE films after 40 days of incubation. The control film was incubated in MSM without microbial inoculation (Control-40d). Experimental groups included films incubated with *Qipengyuania citrea* SCSIO 80490, *Gordonia mangrovi* SCSIO 80507, *Penicillium oxalicum* SCSIO 81005, *Penicillium aethiopicum* SCSIO 81068, and *Streptomyces pluricolorescens* SCSIO 80745. (**A**) FTIR spectra of PE films incubated with different microbial strains compared to the blank control. (**B**) Quantitative comparison of normalized intensity ratios of characteristic absorption bands relative to the 1,460 cm⁻¹ reference band. Notable changes were detected at 720 cm⁻¹ and 730 cm⁻¹ (CH₂ rocking, crystallinity-related), 2,848 cm⁻¹ and 2,915 cm⁻¹ (CH₂ stretching), and in the oxidation-sensitive regions at 1,640 cm⁻¹–1,660 cm⁻¹ (carbonyl index, CI) and 1715 cm⁻¹–1740 cm⁻¹ (vinyl index, VI), indicating chain scission, oxidative modifications, and structural rearrangements of PE films following microbial treatment.

To further verify the underlying chemical changes, FTIR analyses with quantitative evaluation of baseline-corrected peak areas were performed (Fig. 7). Compared to the blank, the characteristic PE peaks at 720 cm⁻¹ and 730 cm⁻¹ (assigned to –(CH₂)n– rocking vibrations) decreased by 5%–14% and 9%–20%, respectively, indicating partial disruption of the crystalline regions of PE. The methylene stretching peaks at 2,848 cm⁻¹ and 2,915 cm⁻¹ also showed reductions of 3%–17%, consistent with chain scission of the PE backbone. More importantly, new absorption bands were detected in the 1,640 cm⁻¹–1,660 cm⁻¹ region (carbonyl index) and 1,715 cm⁻¹–1,740 cm⁻¹ region (vinyl index), both absent in the blank but present in all degraded samples. The carbonyl index reached values of 0.32–1.27, while the vinyl index reached up to 0.74 (strain 80507), providing evidence of oxidative degradation and the introduction of unsaturated structures during microbial attack. Among the identified PE degraders, two strains belong to the genus *Penicillium*, which is known for its abundance of hydrolases and oxidases. Several *Penicillium* species have been reported to degrade various plastics, including PE and PP (46). Additionally, *Gordonia* is also recognized as a promising genus for degrading both C-C and C-O type plastics. For instance, *Gordonia polyisoprenivorans* was the dominant species in a 2-year enrichment study using hexadecane and demonstrated the ability to degrade PE (47). *Gordonia* sp. CN2K has also been shown to degrade PET (48). However, highly efficient degraders for C-C type plastics remain scarce. The five strains identified in this study may contribute to the treatment and recycling of C-C type plastics. Their unique degradation abilities highlight the potential for biotechnological applications in addressing plastic pollution.

### Conclusion

This study provides a rich microbial resource and methodological frameworks that advance our understanding of marine plastic biodegradation. We established the largest culture collection to date of marine-derived plastic-degrading microorganisms, comprising 1,377 bacterial and 202 fungal strains, which provides a foundation for both mechanistic studies and applied bioremediation. Through systematic evaluation of isolation approaches, we showed that selective enrichment combined with carbon-supplemented dual-layer plating enables efficient isolation of plastic-degrading pure cultures. Functional screening revealed phylogenetically specialized degraders, with *Bacillus altitudinis* emerging as the predominant lineage among PU degraders, accounting for 41% of isolates. SEM and FTIR analyses confirmed the efficiency of degraders targeting various polymers, including five PET- and five PE-degrading strains spanning diverse bacterial and fungal taxa such as *Penicillium*, *Streptomyces*, and *Cladosporium*. Collectively, the strain collection and the methodological frameworks developed here provide not only a genetically diverse microbial reservoir but also effective strategies for systematically isolating and evaluating plastic-degrading microorganisms from marine environments. These advances lay both biological and technical foundations for the development of tailored microbial consortia and for promoting sustainable strategies in plastic waste management.

### Highlights

- Landscape of culturable marine plastic-degrading microorganisms identifies 1,377 bacterial and 202 fungal strains, establishing the largest pure-culture collection for plastic biodegradation.
- First systematic methodological benchmarking identifies selective enrichment coupled with carbon-supplemented dual-layer plating as the optimal strategy for marine plastic-degrading strain isolation.
- Plastic-type-dependent culturing yields phylogenetically distinct degraders, with *Bacillus altitudinis* emerging as the dominant polyurethane-degrading taxon.
- Five promising strains for polyethylene terephthalate degradation, 130 for PUR, and 5 for polyethylene were identified, respectively.

## Data Availability

All sequences used for phylogenetic reconstruction have been deposited in the NCBI database under accession numbers PX361377 to PX362603 for bacterial 16S rRNA genes and PX360325 to PX360525 for fungal ITS sequences.

## AFTER EPUB

*[This article was published on 8 December 2025 with errors in two strain names. The names were corrected in the current version, posted on 20 January 2026.]*

## SUPPLEMENTAL MATERIAL

Supplemental figures msystems.01388-25-s0001.docx Fig. S1 to S6.

Supplemental tables msystems.01388-25-s0002.xlsx Tables S1 to S11.

ASM does not own the copyrights to Supplemental Material that may be linked to, or accessed through, an article. The authors have granted ASM a non-exclusive, world-wide license to publish the Supplemental Material files. Please contact the corresponding author directly for reuse.
