# Supplementary material for: Isolation and characterization of marine microorganisms capable of degrading plastics
Source: mSystems. 2025 Dec 8;11(1):e01388-25. doi: 10.1128/msystems.01388-25 (PMC12817902; doi:10.1128/msystems.01388-25)
Supplement: Supplemental figures — Fig. S1 to S6. [file msystems.01388-25-s0001.docx]

**Isolation and Characterization of Marine Microorganisms Capable of Degrading Plastics**

Qi Zeng^1,2^, Liwen Chang^1,2^, Yuqing Liu^1,2^, Songbiao Shi^1^, Jian Yang^1^, Qinglian Li^1,2^, Lijuan Long^1^, Xinpeng Tian^1*^

^1^ State Key Laboratory of Tropical Oceanography; Key Laboratory of Tropical Marine Bio-resources and Ecology; Guangdong Key Laboratory of Marine Materia Medica; RNAM Center for Marine Microbiology; South China Sea Institute of Oceanology, Chinese Academy of Sciences, Guangzhou, Guangdong 510301, China

^2^University of Chinese Academy of Sciences, Beijing 100049, China

*****Correspondence: for correspondence: Xinpeng Tian; Tel / Fax: +86 20 89023378; E-Mail: xinpengtian@scsio.ac.cn;


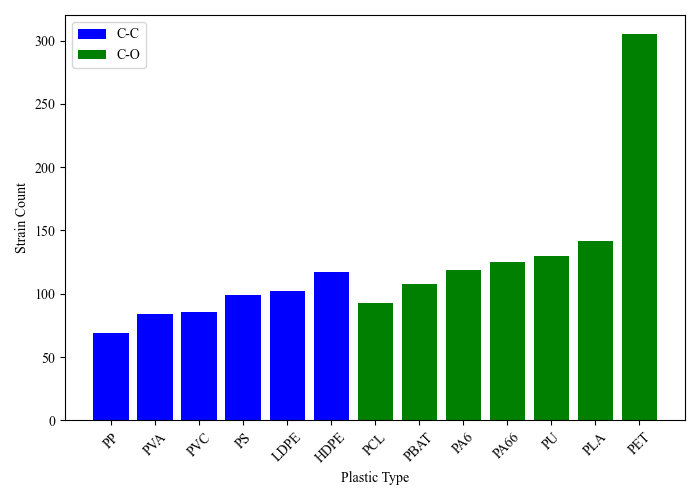


**Fig. S1 Number of microbial strains isolated from different plastic types.** Bar chart showing the distribution of strains isolated from 13 types of plastic plates. Plastics with carbon–carbon (C–C) backbones are shown in blue (PP, PVA, PVC, PS, LDPE, HDPE), while those with carbon–oxygen (C–O) backbones are shown in green (PCL, PBAT, PA6, PA66, PU, PLA, PET).


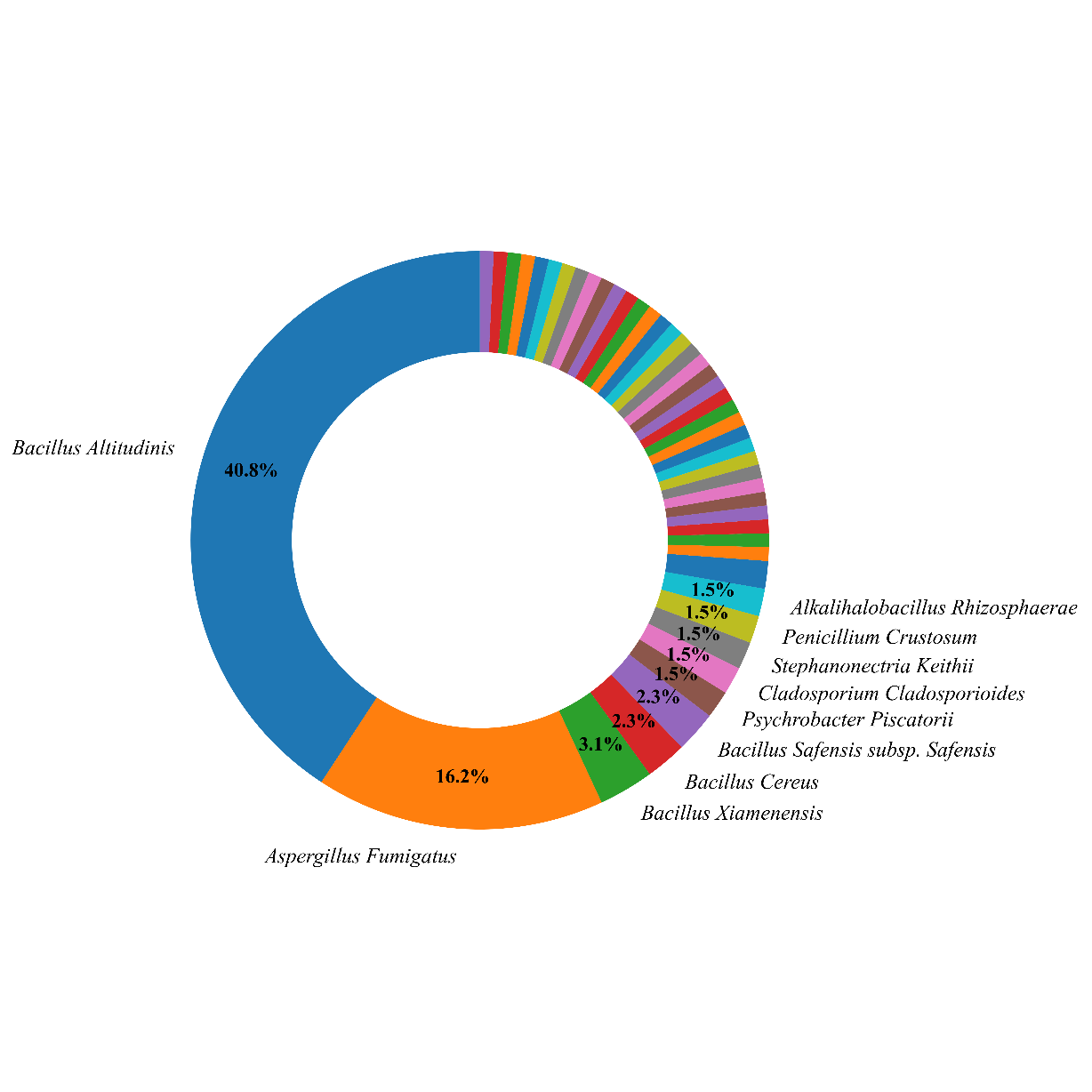


**Fig. S2** The taxonomic diversity and relative abundance of isolates exhibiting clear zones on polyurethane (PU) plates. Only the ten most abundant species are labeled.


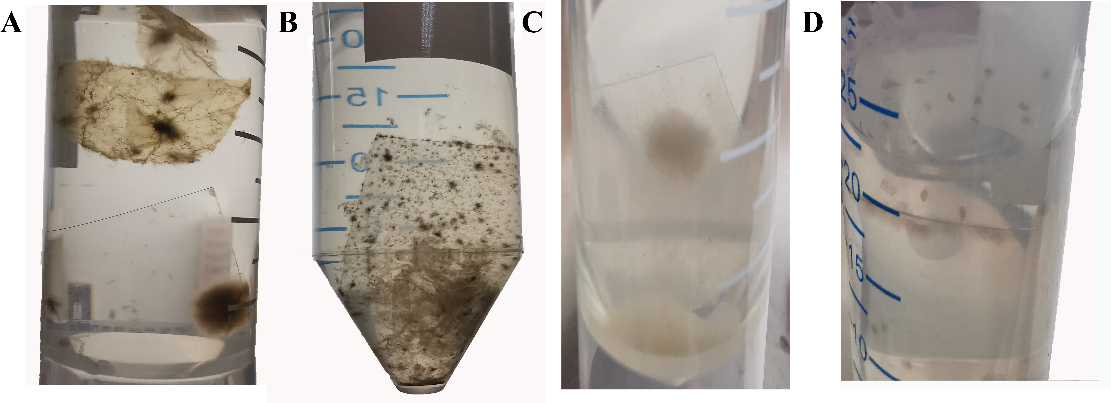


**Fig. S3 Microbes adhere and grow on plastic film during enrichment.** (A) ST1 sample was enriched with PET film as the main carbon source (B) H2 sample was enriched with PVC film as the main carbon source (C) H4 sample was enriched with PET film as the main carbon source (D) H2 sample was enriched with PP film as the main carbon source


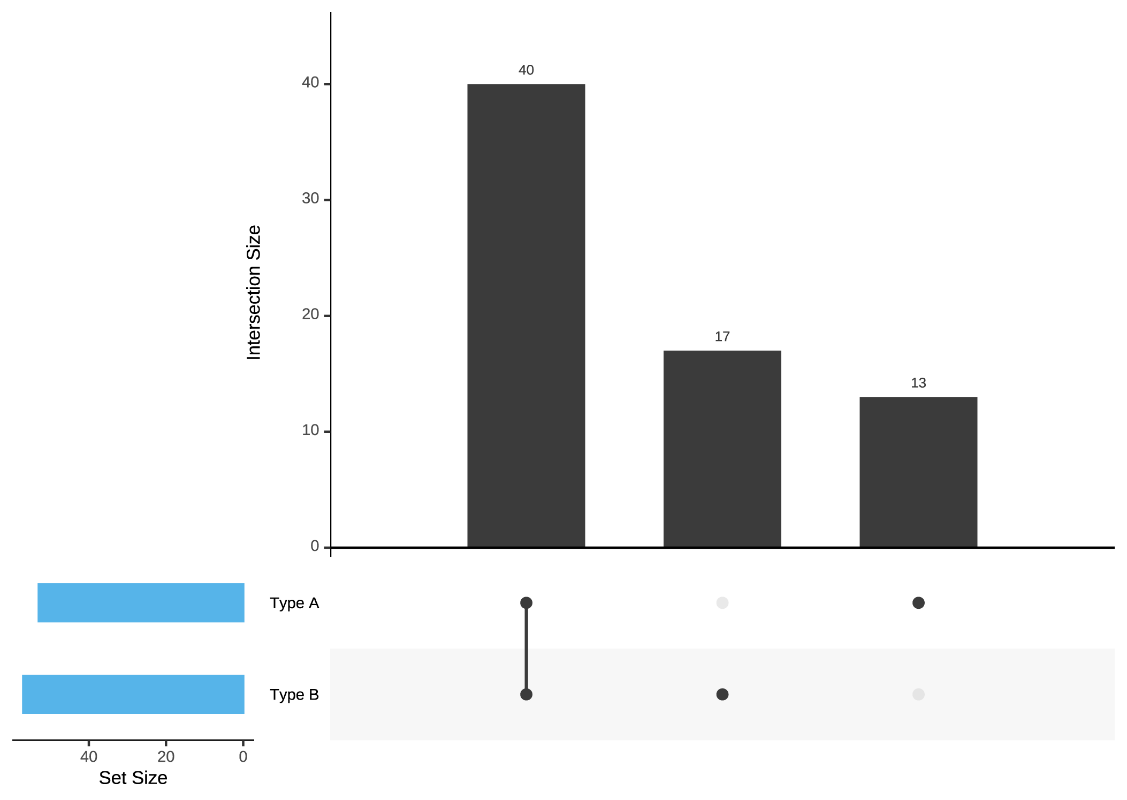


**Fig. S4 Comparison of genera isolated using different media.** Type A medium consisted of MSM supplemented with plastic as the sole carbon source, while Type B medium contained an additional basal layer of 2216E. The UpSet plot shows the number of genera shared between both media (intersection, n = 40) as well as those uniquely recovered from Type A (n = 17) or Type B (n = 13).


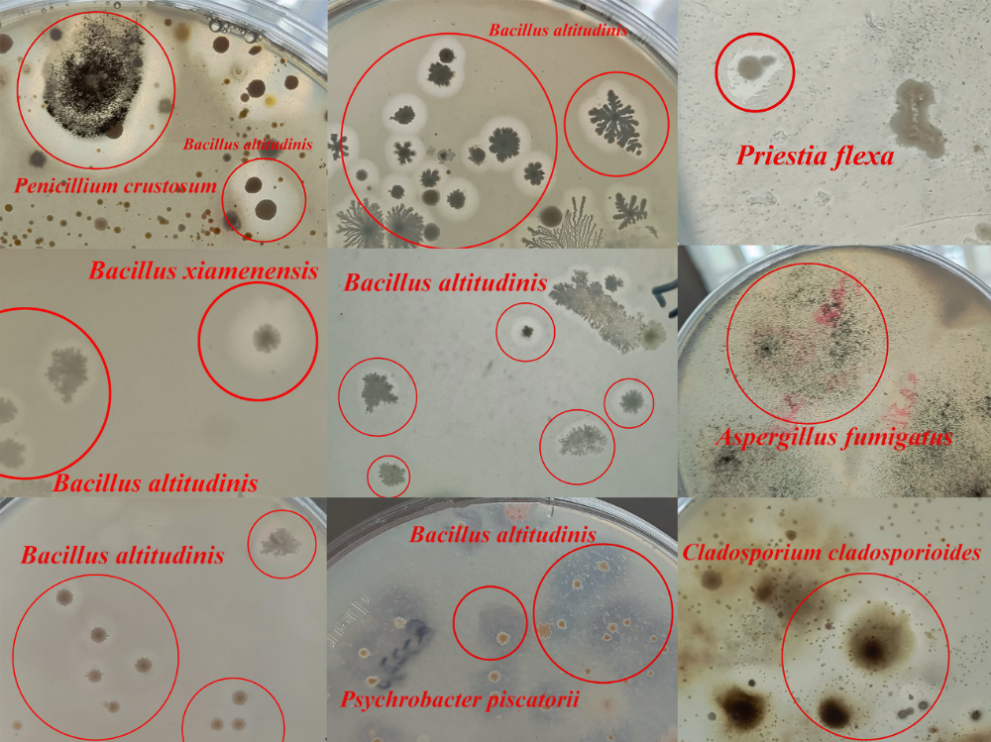


**Fig. S5 Microorganisms Producing Clear Zones on Polyester-Based PU (Impranil™ DLN) Plates.** Representative images of bacterial and fungal strains showing hydrolytic activity toward Impranil™ DLN, as indicated by the formation of clear zones surrounding colonies (highlighted in red circles).


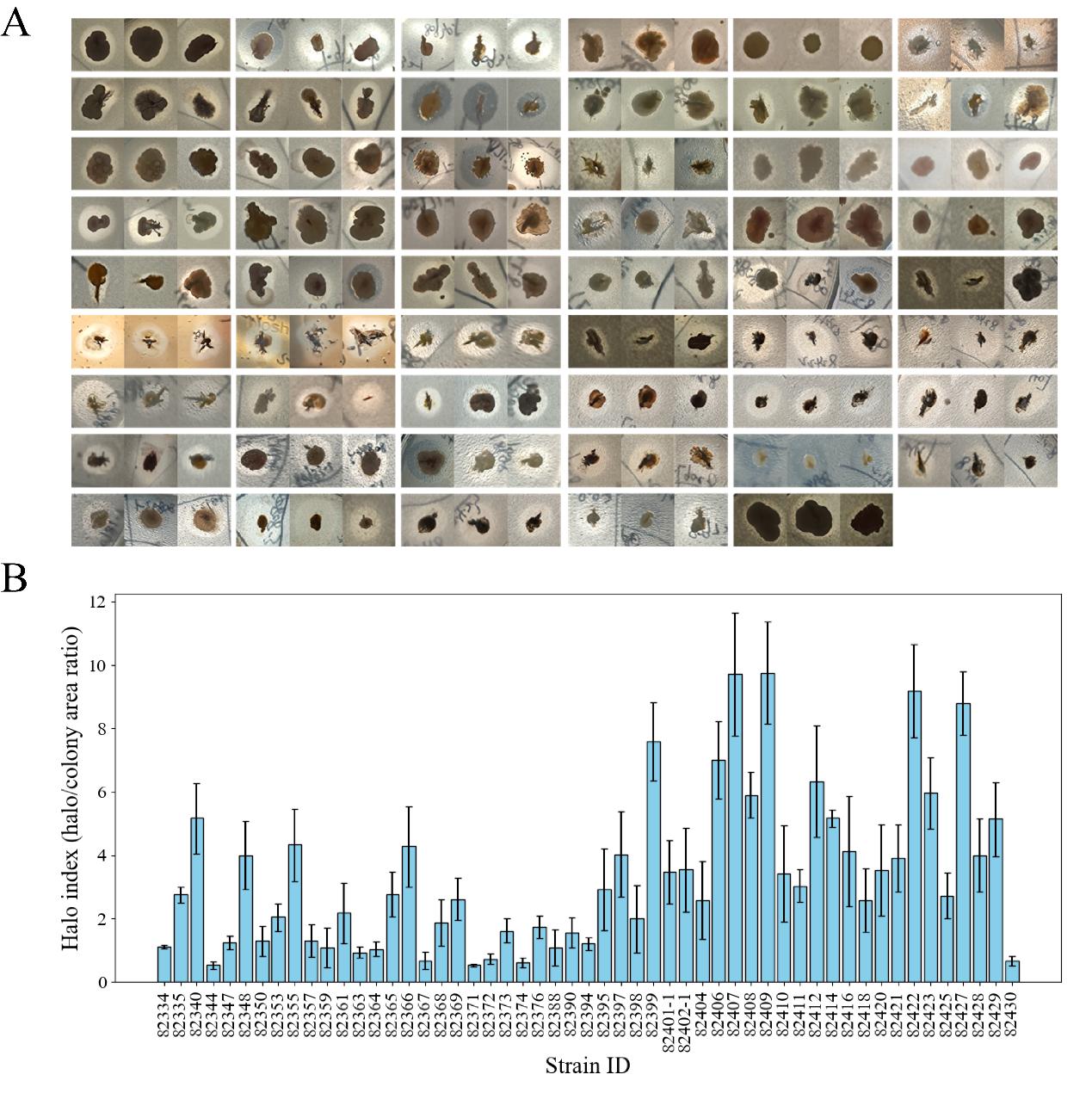


**Fig. S6 Polyurethane (PU) degradation activity of *Bacillus altitudinis* isolates.** (A) Representative photograph showing transparent halo formation around colonies grown on Impranil™ DLN agar plates (MSM with 3% PU and 0.5% peptone), indicating enzymatic degradation of PU. (B) Quantitative comparison of 53 *B. altitudinis* isolates, expressed as the halo index (HI), i.e., the ratio of transparent zone area to colony area. Each bar represents the mean of three independent replicates, and error bars indicate standard deviation (SD).
